# Evaluation of the Antimicrobial Effect of Thymoquinone against Different Dental Pathogens: An In Vitro Study

**DOI:** 10.3390/molecules26216451

**Published:** 2021-10-26

**Authors:** Khalifa S. Al-Khalifa, Rasha AlSheikh, Moahmmed T. Al-Hariri, Hosam El-Sayyad, Maher S. Alqurashi, Saqib Ali, Amr S. Bugshan

**Affiliations:** 1 Department of Preventive Dental Sciences, College of Dentistry, Imam Abdulrahman Bin Faisal University, Dammam 31441, Saudi Arabia; 2 Department of Restorative Dental Sciences, College of Dentistry, Imam Abdulrahman Bin Faisal University, Dammam 31441, Saudi Arabia; ralsheikh@iau.edu.sa; 3 Department of Physiology, College of Medicine, Imam Abdulrahman Bin Faisal University, Dammam 31441, Saudi Arabia; mtalhariri@iau.edu.sa; 4 Middle Eastern Regional Radioisotope Centre for the Arab Countries, Giza 12311, Egypt; hoselsayyad@gmail.com; 5 Department of Microbiology, College of Medicine, Imam Abdulrahman Bin Faisal University, Dammam 31441, Saudi Arabia; msalqurashi@iau.edu.sa; 6 Department of Biomedical Dental Sciences, College of Dentistry, Imam Abdulrahman Bin Faisal University, Dammam 31441, Saudi Arabia; samali@iau.edu.sa (S.A.); abugshan@iau.edu.sa (A.S.B.)

**Keywords:** black seeds, thymoquinone, dental caries, antibacterial agents, herbs

## Abstract

This study aimed to evaluate the antimicrobial effect of Thymoquinone (TQ) on four different oral microorganisms. Minimum Bactericidal Concentration (MBC), Minimum Inhibition Concentration (MIC), Broth microdilution, and Well diffusion tests were used to determine the optimum antimicrobial concentrations of TQ against *Streptococcus salivarius, Streptococcus oralis, Streptococcus mutans,* and *Staphylococcus aureus* over 1, 3, 6, 12 and 24 h. Chlorhexidine 0.12% was selected as a positive control. The inhibitory effect of TQ on bacterial growth was most noticeable with *S. salivarius,* while the least affected was *S. aureus.* TQ’s MBC and MIC for *S. oralis* and *S. aureus* were comparable 2 mg/mL and 3 mg/mL, respectively. *S. salivarius* was most resistant to TQ and displayed a value of 5 mg/mL and 4 mg/mL for MIC and MBC, respectively. The viable count of different strains after exposure to TQ’s MBC values was most noticeable with *S. aureus* followed by *S. oralis* and *S. mutans,* while *S. salivarius* was least affected. This study emphasized the promising antimicrobial effect of TQ against the four main oral microorganisms. It has a potential preventive effect against dental caries as well as other oral diseases.

## 1. Introduction

The oral cavity is inhabited by hundreds of microorganisms of different species, mainly bacteria, forming the oral biofilm. The formation of the biofilm and the type of microorganisms are influenced by numerous host and environmental factors. Biofilm formation in the oral cavity can cause multiple different diseases depending on the prominent microorganisms, such as caries, root canal infections, periodontal diseases, and peri-implant diseases, and other gastric intestinal track-related diseases [1,2,3].

Dental caries is an infectious disease that starts with a bacteria-colonized dental plaque. Dental plaque is a sticky, highly hydrated extracellular biofilm that forms on the tooth surface then later is colonized by cariogenic bacteria that ferment dietary carbohydrate-producing acid. The acid then dissolves tooth mineral content causing cavitation, known as dental caries [4]. It is a multifactorial disease that represents a significant public health challenge worldwide [5]. Despite all the advancements in dental education and oral hygiene awareness, dental caries remains the leading health challenge in most developed countries, where 60–90% of children and most adults suffer from dental caries. On the contrary, developing countries have caries prevalence reported to reach as high as 68% [6]. The latest global reports noted that untreated dental caries is still a common disease worldwide [6]. Recent studies in different countries around the world indicated an increase in caries’ prevalence [7,8]. Caries lesions can be asymptomatic in their initial stages. In contrast, advanced caries lesions can cause toothache, which adversely affects life quality by affecting the mastication efficiency, patient smile, and self-consciousness. Children suffering from caries suffer relatively from poor nutritional health, poor child growth, and low weight gain [9].

Although several different bacteria have been associated with dental caries’ pathogenesis, *Streptococcus mutans* is the most relevant acidogenic-aciduric bacterial species. It plays the primary role in initiating dental caries lesions [10,11]. Other cariogenic bacteria are responsible for the sustainability and progression of the caries lesion. Studies investigating the prevalence of cariogenic bacteria and other bacterial microorganisms in caries lesions revealed a high prevalence of *S. mutans*, *S. salivarius*, and *S. oralis* in samples collected from active supra gingival caries lesions [2,3]. *S. aureus* is associated with dental implant infection and has established a high tolerance to common antimicrobial treatments [1].

The use of herbs and plant extract by humans as medicine is an ancient practice that goes back thousands of years. Lately, medicinal plants have been experiencing a growing popularity and interest due to the public concern with the adverse effect of synthetic drugs [12,13]. Plant extract medicine is being widely investigated and proven to have exceeded expectations in preventing and treating a wide array of diseases and medical conditions [14,15].

Black cumin (*Nigella sativa*), belonging to the Ranunculaceae family, has been used historically by various cultural traditional medicinal treatments. Traditionally it has been used to treat various diseases, such as headaches, influenza, dyspepsia, diabetes, and asthma [16]. Black seed extract has been confirmed to improve oral health and reduce dental caries, [17] periodontitis, gingivitis, and pulp diseases [18].

Black seed extract includes essential oils, fixed oils, alkaloids, saponins, and proteins. Thymoquinone (TQ) is the core ingredient of the black seed oil extract with proven medical benefits [19]. The literature suggested a significant decrease in the count of *S. mutans* when exposed to TQ [17].

Most published studies focused on the antibacterial effect of TQ against a single strain of bacteria or by using one method of evaluation. There have not been any extensive studies exploring the antibacterial effect of TQ on several bacterial strains. Hence, this study aimed to evaluate the antimicrobial effect of the TQ (concentration and exposure duration) on four different oral microorganisms. The tested hypothesis states that there is no difference in bacterial growth when using the TQ solution’s different concentrations with the control groups (negative and positive).

## 2. Results

The antimicrobial potential of TQ at different concentrations compared to complimentary control CHX mouth wash is shown in Table 1. The findings indicated that the microbial activity of TQ was concentration-dependent. Interestingly, there was no bacterial growth for TQ concentrations of 75 and 100 mg/mL. The inhibitory effect of TQ on bacterial growth was most noticeable with *S. salivarius* followed by *S. mutans* and *S. oralis,* and the least affected was *S. aureus.*

**Figure 1:**
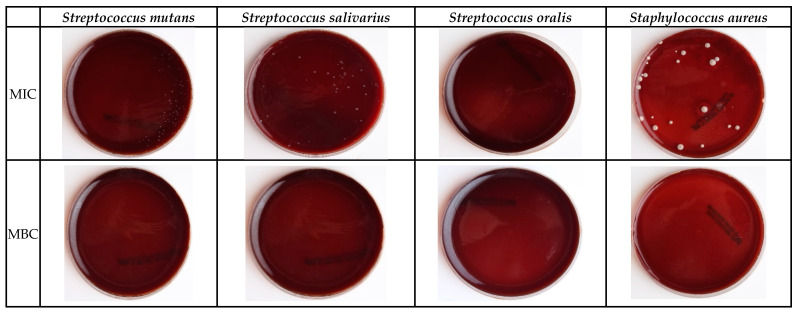
Bacterial growth; MIC (Minimal Inhibitory Concentrate) and MBC (Minimal Bactericidal Concentrate) of TQ with the four bacterial strains.

To ensure TQ’s antibacterial effect, colonies of the four bacterial strains were counted after applying the same TQ doses using cell culture counts to estimate the MIC and MBC (Figure 1). TQ’s minimum concentration required for inhibition (MIC) or killing (MBC) of *S. oralis* and *S. aureus* was comparable 2 and 3 mg/mL, respectively, as they were the most sensitive to TQ. On the contrary, *S. salivarius* showed the highest resistance to TQ with the values of 5 and 4 mg/mL for MBC and MIC, respectively. TQ’s minimum concentration required for inhibition (MIC) and killing (MBC) of *S. mutans* was 3 and 4 mg/mL, respectively.

On the other hand, CHX exhibited better antibacterial activity when compared to TQ; presented MBC ≥ 0.0009 mg/mL (≥0.00009%), and MIC ≥ 0.00002 mg/mL (≥0.000002%) for the microorganisms tested (Table 2).

**Figure 2:**
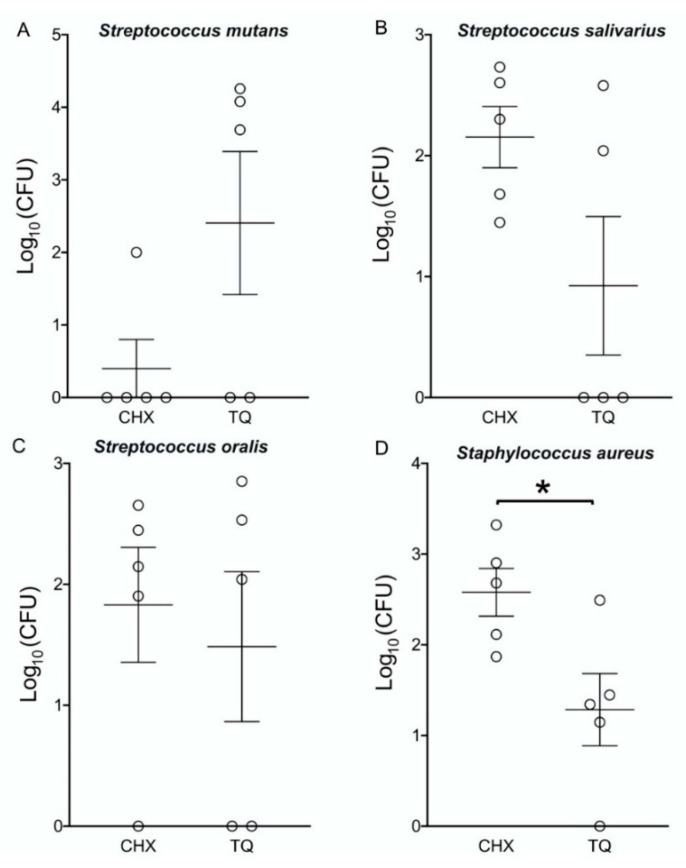
Viable bacterial count using Log10 transformation for CHX (chlorohexidine) TQ (Thymoquinone). * Statistically significant difference at (*p* < 0.05).

The viable count of different strains after exposure to TQ’s MBC values was most noticeable with *S. aureus* followed by *S. oralis* and *S. mutans*, the least affected was *S. salivarius.* A significant drop over time in CFU counts was noted, except at 24 h, where no difference was detected (Table 3). The Log10 transformation results showed only a statistically significant difference between TQ and CHX with the *S. aureus* bacterial strain. The colony-forming units (CFU) of the different bacterial strains after exposure to the minimal bactericidal doses of TQ and the corresponding doses of the control chlorhexidine (CHX) for each bacterial strain. The dot-plot shows the distribution of the individual measurements and the mean (horizontal line), and the standard error of the mean (capped vertical line). A statistically significant difference (*p* < 0.05) is indicated with bars and an asterisk (Figure 2).

## 3. Materials and Methods

### 3.1. Thymoquinone (TQ) Preparation

Dry powder extract of TQ (Sigma-Aldrich) and TQ oil was obtained and used in the experiments. Five hundred mg of TQ was dissolved in 1 mL of methanol. The tested compounds were diluted in brain heart infusion broth (BHIB) to prepare seven different concentrations (0.2, 0.5, 1.0, 1.5, 2.0, 3.0 and 4.0 mg/)mL and two control test tubes, one negative control (1ml brain-heart fusion broth without TQ) and one positive control (1 mL (0.12%) Chlorohexidine; Avohex, Middle East Pharmaceutical Industries Ltd., Riyadh, Saudi Arabia) [20,21].

### 3.2. Microorganism

Four Gram-positive strains were used in this research, *S. mutans* (ATCC 25175)*, S. aureus* (ATCC 25923), *S. oralis* (ATCC 6249), and *S. salivarius* (ATCC 13419), obtained from Microbiologics, St. Cloud, MN, USA, and cultivated on Mueller-Hinton Agar with 5% Sheep Blood (HiMedia Laboratories Pvt. Ltd., Mumbai, India) for 48 h at 37 °C, with 5% CO2. After this period, bacteria colonies were picked up from the new culture and suspended in 2 mL of sterile distilled water, then serial diluted (fivefold) from 1:10 to 1:10^5^.

### 3.3. Antibacterial Activity Assay of Thymoquinone (TQ) against Tested Organisms

This study used seven different concentrations of TQ, where each test tube containing 1 mL of sterile BHIB with different concentrations of TQ separately (0.2, 0.5, 1.0, 1.5, 2.0, 3.0 and 4.0 mg/mL) and 0.1 mL of tested organisms. Furthermore, 0.1 mL of tested organisms were mixed with 0.9 mL sterile BHIB as a negative control. The positive control was 0.1 mL of tested organisms added to 0.9 mL chlorohexidine. Then 250 µL from each concentration was cultured on Mueller-Hinton Agar with 5% Sheep Blood and then incubated for 48 h at 37 °C in a microaerophilic condition (10% CO_2_) candle gar method for *S. mutans, S. oralis*, and *S. salivarius* and with aerophilic conditions for *S. aureus*. Triplicates of the TQ, as well as the control solution, were performed in this study.

### 3.4. Minimum Inhibitory Concentration (MIC) Test

After 24 h of incubation, the number of colonies forming units per millimeter was determined for each concentration to detect the minimum inhibitory concentration (MIC) by the lowest TQ concentration that inhibits bacteria growth. The MIC of Thymoquinone (TQ) against different test bacteria was determined by broth microdilution assay growth using the clinical and laboratory standards institute guidelines [22].

### 3.5. Minimum Bactericidal Concentration (MBC) Test

From all MIC test tubes that showed no growth, 250 μL of culture was inoculated into fresh Mueller-Hinton Agar with 5% Sheep Blood plates. After incubation for 48 h under suitable conditions according to the tested microorganism, as previously described, the minimum bactericidal concentration (MBC) was determined by observing bacterial growth. The concentration of antimicrobial agents that eliminate more than 99.9% after 24 h of incubation was recorded as MBC.

The following equation calculated the reduction of bacterial count percentage for each concentration:

(1) $$\mathrm{Reduction}\;\mathrm{of}\;\mathrm{bacterial}\;\mathrm{count}\;\%\;=\frac{C\;\mathrm{control}-C\;\mathrm{sample}}{C\;\mathrm{control}}\times 100$$

After 48 h of incubation, the colonies were counted.

### 3.6. The Bacterial Count of MBC Values during Different Periods

The MBC value of each tested organism for TQ was inculcated with bacterial inoculums (1.7 × 10^5^ CFU/mL) in sterile Eppendorf containing 1 mL of BHIB. Furthermore, tested organisms were mixed with one ml of positive control (Chlorhexidine). All tested groups and control were incubated for 24 h at 37 C. Then, 0.1 mL of each group was assessed periodically after 1, 3, 6, 12 and 24 h from inoculum by preparing serial dilutions in a sterile saline solution (0.9%). After that, 250 µL from these dilutions was added and spread on Mueller-Hinton Agar with 5% Sheep Blood in duplicate and incubated under suitable conditions according to the tested microorganism, as previously described.

### 3.7. Well Diffusion Test

The well diffusion method was performed by using blood Muller-Hinton agar (BMHA) [23]. The BMHA plate surface was inoculated for a defined volume of all test isolates using a sterile cotton swab over the entire plate surface. A hole (6 mm in diameter) was cut in the agar gel, and then 100 µL of five various concentrations of TQ (12.5, 25, 50, 75 and 100 mg/mL) were added to each well, separately. Chlorohexidine was used as a positive control. Then, all plates were incubated under suitable conditions according to the tested microorganism for 24 h. All experiments were performed in duplicates for each antimicrobial agent. After the incubation period, bacterial growth inhibition (the endpoint of the clear zone diameter) was measured (mm).

## 4. Statistical Analysis

The obtained CFU data were pooled for the different time points and processed with Log10 transformation. The comparisons between test (TQ) and control [CHX] were performed, for each bacterial strain, using an independent *t*-test. The statistical analysis was performed in IBM SPSS^®^ Statistic software (Version 25). The CFU results are provided in dot-plots, showing the individual measurements and the mean and the standard error of the mean. The microbial inhibition zone results and the minimum inhibitory and bactericidal concentrations are provided descriptively in tables.

## 5. Discussion

Dental caries persists to be a worldwide health problem despite clinical and medical advancement. The cariogenic bacteria colonize the dental plaque to induce the disease; different strategies to inhibit the bacterial growth and prevent colonization have been investigated and later implemented to control dental caries. Cariogenic bacteria have been linked to several systemic diseases, such as infective endocarditis, ulcerative colitis, peritonitis, and atherosclerosis [24,25]. Recently, natural products have been experimentally investigated to inhibit the growth of dental plaque microorganisms [12]. In addition to the public interest in organic natural products, the ease of extraction and large-scale production render such products more alluring to manufacturers [26].

The medicinal uses of the *Nigella sativa* have been well documented over the years. The literature has proven the effectiveness of TQ, its active ingredient, against various pathologies, such as cancer, inflammation, allergies, and microbial [27,28,29,30].

Minimal inhibitory concentrate (MIC) and the minimal bactericidal concentration (MBC) are considered the golden standards to evaluate the antimicrobial effect of an agent against microorganisms, and as such, is used to judge other methods to evaluate antimicrobials [31].

In the current study, TQ displayed promising results in inhibiting the growth of oral bacterial strains. The null hypothesis was rejected as the TQ was most potent against *S. oralis, S. mutans* showed an MIC of 3mg/mL and MBC of 4mg/mL, whereas *S.* *salivarius* showed most resistance, with an MIC of 4 mg/mL and MBC 5 mg/mL of TQ. All four species showed the same resistance pattern when using the control chlorohexidine CHX, where both *S. aureus* and *S. oralis* showed the least resistance and *S. salivarius* showed the most resistance. *S. mutans* is one of the main bacteria that initiate dental caries. In previous studies, the presence of *S. salivarius* in a high number in active carious lesions helps sustain and progress carious lesions [12,13,14,25]. Our results are in line with other studies showing bactericidal, as well as growth inhibition, against *S. mutans* [32]. *S. oralis* is also one of the bacteria found in the active carious lesion. They are also considered to take part in the progression and the sustainability of the caries process [15,16,27]. These results were consistent with the study on different bacteria strains, showing *S. salivarius, S. mutans,* and *S. aureus* sensitivity to TQ, with MIB and MIC values being significant [33,34].

Another interesting finding of this study was that there was no microbial activity/growth when TQ concentrations were 75 and 100 mg/mL. Previous studies have stated that TQ can be toxic at a certain concentration that starts from 92%. The concentration which showed no microbial activity is still below the toxic concentration [34]. It can be reported that the microbial activity of TQ is concentration-dependent. *S. aureus and S. oralis* showed maximum inhibition at a TQ concentration of 25 mg/mL, followed by *S. mutans* at 50 mg/mL, then *S. salivarius* at 75 mg/mL. A previous study has shown dose-dependent antibacterial activity, which is in line with our results. It also reported it better against gram-positive in comparison with gram-negative bacteria. *S. aureus* exhibited the highest sensitivity to TQ amongst the different gram-positive, and gram-negative species tested [35]. *S. aureus* is one of the most common pathogens that is faced in clinical settings. Aside from this, *S. aureus* is found in infections related to dental implants, and it is of utmost importance, as infection can decide the faith of success of the dental implant. Our result is in line with the previous results, showing antibacterial activity, minimum inhibitory effect, and being more tolerant than the rest of the three, *S. oralis, S. mutans* and *S. salivarius*, bacterial strains [36,37]. While *S. aureus* has shown resistance against common antimicrobial treatments, an increase in the dose of TQ has been effective in reducing the bacterial count and growth; TQ could be the answer to overcoming this strand’s high resistance [17,18,28].

To further validate the study findings against the standard laboratory testing, a well diffusion test was carried. The TQ inhibition effect is not only concentration-dependent but also time-dependent; for *S. oralis, S. salivarius,* as well as *S. aureus.* TQ proved to be faster than the CHX control in stopping the growth of bacteria. While *S. salivarius* and *S. aureus* were still present after 24 h when using CHX; with TQ, *S. salivarius* was eliminated after 6 h and *S. aureus* after 12 h. Other studies reflected similar findings; Mouwakeh et al. stated they had more potent inhibitory activity over time against *S. oralis* [38].

Such finding suggests that the use of TQ in low concentrations can be effective against caries progression, as well as peri-implantitis, which is the severe inflammation around dental implants. While higher concentrations can be beneficial to caries initiation prevention. While CHX can be providing the same benefits as TQ, the latter has shown a promising advantage over CHX, being effective in short periods.

Other studies have focused on MIC or MBC [32,33], others on bacterial inhibition zone [35,39], or limit the study to one or two bacterial strains. This study’s strength is that it covered a more comprehensive selection of cariogenic bacteria, caries initiation, and caries sustaining and progression enhancing microorganisms. Simultaneously, covering the MIC, MBC, and the inhibitory zone are viable tests in studying experimental agents’ antibacterial activities.

Other essential oils have been investigated and proven effective in dental plaque control and reducing the count of cariogenic microorganisms. Filogônio et al. added mineral Brazilian nut oil to the commercially available dentifrice. He stated that after 90 days, the tested oils were sufficient to improve dental biofilm control significantly [40]. Lobo et al. compared the effectiveness of Lippia sidoides Cham essential oil (LSO) to CHX in reducing the salivary *S. mutans* in children with caries. In contrast, CHX reduces *S. mutans* after using the mouthwash, but the *S. mutans* count returns to baseline levels shortly after treatment. While using the LSO, the *S. mutans* count was reduced and remained low even after treatment [41]. Charugundla et al. investigated the essential oil in reducing dental plaque compared to CHX and Fluoride mouth rinse; all three mouth rinses significantly reduced plaque accumulation and gingivitis, particularly in caries-free subjects against those with caries. [42]

This study evaluated TQ’s antibacterial effect against four potent bacteria essential to initiate and sustain caries lesions. However, dental caries starts with dental plaque, which exists in the oral cavity in a biofilm. Further clinical studies to evaluate the effect against other biofilm bacteria and in vitro evaluation of the TQ antibacterial potential are recommended. Further studies with different TQ concentrations and intervals, combined with other essential oils that can modify or boost its effect, can be of great benefit to explore TQ’s therapeutic and preventive role.

## 6. Conclusions

The inhibitory effect of TQ on bacterial growth was most noticeable with *S. salivarius* followed by *S. mutans* and *S. oralis,* as the least affected was *S. aureus.* In addition, TQ’s MIC and MBC against the different bacterial strains were tested, and the results were significant. This study emphasized the promising cariogenic antimicrobial effect of TQ against the four main oral microorganisms, not only that TQ exhibits an antimicrobial effect comparable to standard antimicrobial medicine with a longer duration effect. It has a potential therapeutic and preventive effect against dental caries as well as other oral diseases. A significant highlight is eliminating chemically synthesized drugs and the growing concerns of bacterial resistance and medical side effects.

## Figures and Tables

**Table 1:** Microbial inhibition zone in millimeters provided by TQ at different concentrations compared to a positive control (CHX).

Concentration (mg/)mL	Inhibition Zone (Mean and SD in mm)			
	*S. salivarius*	*S. mutans*	*S. oralis*	*S. aureus*
12.5	37 ± 1.5	29.5 ± 1.8	41.5 ± 1.5	36.5 ± 1.5
25	38.5 ± 1.5	37.5 ± 1.1	44 ± 0.0	44 ± 0.0
50	38.5 ± 1.0	44 ± 0.0	44 ± 0.0	44 ± 0.0
75	44 ± 0.0	44 ± 0.0	44 ± 0.0	44 ± 0.0
100	44 ± 0.0	44 ± 0.0	44 ± 0.0	44 ± 0.0
CHX (+ve control)	11.5 ± 1.0	17.5 ± 0.2	21.5 ± 1.5	12 ± 1.0

Note: The plate diameter is 88 mm (half diameter = 44), which designates no bacteria growth.

**Table 2:** Minimum Inhibitory and Bactericidal Concentration (mg/mL) of TQ and positive control CHX against the four bacterial strains.

Microorganisms	TQ		CHX	
	MIC	MBC	MIC	MBC
*S. salivarius*	4.0	5.0	0.00857	0.0171
*S. mutans*	3.0	4.0	≤0.00002	0.0009
*S. oralis*	2.0	3.0	0.00095	0.0038
*S. aureus*	2.0	3.0	0.00857	0.0171

**Table 3:** The viable count of different strains after exposure to the MBC values of TQ and positive control (CHX) during different observation periods.

Time (hours)	*S. salivarius*		*S. mutans*		*S. oralis*		*S. aureus*	
	TQ 5 mg/mL	CHX	TQ 4 mg/mL	CHX	TQ 3 mg/mL	CHX	TQ 3 mg/mL	CHX
1 h	0.38 × 10^3^	0.54 × 10^3^	1.8 × 10^4^	1.0 × 10^2^	0.71 × 10^3^	0.45 × 10^3^	0.31 × 10^3^	0.21 × 10^4^
3 h	0.11 × 10^3^	0.40 × 10^3^	1.2 × 10^4^	NG	0.34 × 10^2^	0.28 × 10^3^	0.28 × 10^2^	0.80 × 10^3^
6 h	NG	0.20 × 10^3^	4.9 × 10^3^	NG	0.11 × 10^2^	0.14 × 10^3^	0.22 × 10^2^	0.48 × 10^3^
12 h	NG	0.48 × 10^2^	NG	NG	NG	0.80 × 10^2^	0.14 × 10^2^	0.13 × 10^3^
24 h	NG	0.28 × 10^2^	NG	NG	NG	NG	NG	0.74 × 10^2^

NG: no growth.

## Data Availability

Data sharing not applicable. No new data were created or analyzed in this study. Data sharing is not applicable to this article.

## Sample Availability

Samples of the compounds are not available from the authors.
